# New Evidence for Cross Talk between Melatonin and Mitochondria Mediated by a Circadian-Compatible Interaction with Nitric Oxide

**DOI:** 10.3390/ijms140611259

**Published:** 2013-05-28

**Authors:** Paolo Sarti, Maria Chiara Magnifico, Fabio Altieri, Daniela Mastronicola, Marzia Arese

**Affiliations:** 1 Department of Biochemical Sciences, Sapienza University of Rome, Rome 00185, Italy; E-Mails: mariachiara.magnifico@uniroma1.it (M.C.M.); fabio.altieri@uniroma1.it (F.A.); marzia.arese@uniroma1.it (M.A.); 2 CNR Institute of Molecular Biology and Pathology, Rome 00185, Italy; E-Mail: daniela.mastronicola@uniroma1.it

**Keywords:** nitric oxide, cell bioenergetics, respiratory chain, circadian rhythm, cell culture, Warburg effect, reactive oxygen, nitrogen species

## Abstract

Extending our previous observations, we have shown on HaCat cells that melatonin, at ~10^−9^ M concentration, transiently raises not only the expression of the neuronal nitric oxide synthase (nNOS) mRNA, but also the nNOS protein synthesis and the nitric oxide oxidation products, nitrite and nitrate. Interestingly, from the cell bioenergetic point of view, the activated NO-related chemistry induces a mild decrease of the oxidative phosphorylation (OXPHOS) efficiency, paralleled by a depression of the mitochondrial membrane potential. The OXPHOS depression is apparently balanced by glycolysis. The mitochondrial effects described have been detected only at nanomolar concentration of melatonin and within a time window of a few hours’ incubation; both findings compatible with the melatonin circadian cycle.

## 1. Introduction

Melatonin, the *N*-acetyl-5-methoxytryptamine, is an amphiphilic molecule with remarkable antioxidant properties [1,2]. Originally recognized as the hormone of the pineal gland, melatonin is produced also by other extrapineal sites [3–8]. World-widely recommended as a pharmaceutical tool for elderly people with sleep disorders, melatonin is responsible for regulation of the sleep-wake cycle [9,10]. It is involved in a variety of physiological functions [11–13], including modulation of gene transcription [14], blockage of transcriptional factors [15] and control of mitochondrial activities [16]. The antioxidant properties of melatonin have been world-widely recognized, likely accounting for a number of protective effects exerted in different cellular compartments [17]. Melatonin is more effective than the majority of its naturally occurring molecular analogs [18,19], suggesting that the substituents of the central indole structure controls the reactivity of the adducts. Rate constants determined for the reaction of melatonin with hydroxyl radicals are very high, almost diffusion limited, approaching *k* ≈ 10^10^ M^−1^s^−1^[20,21]. Despite its common use, the molecular mechanism(s) underlying the functional effects of melatonin, particularly those related to cell bioenergetics, remain as yet only partly understood. The blood circulating melatonin concentration is genetically encoded [22] and varies within individuals [23–26], accounting for the existence of genetically encoded melatonin-dependent human syndromes [27].

**Figure 1:**
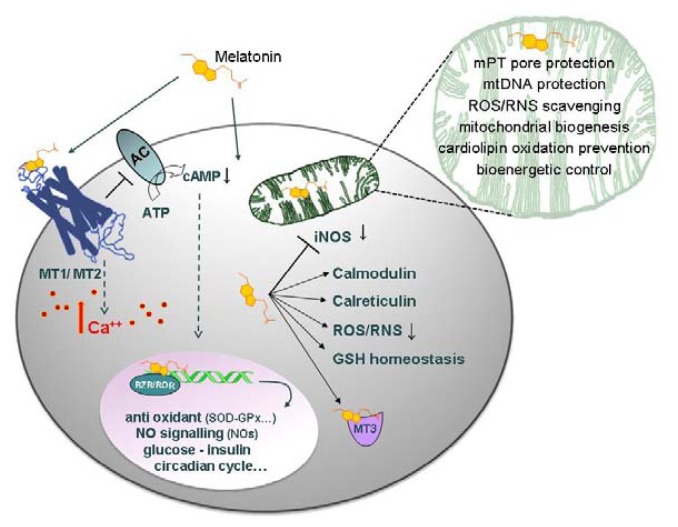
Melatonin on the cell stage. Melatonin interacts with cells in a receptor-dependent or -independent manner. The receptors on the cell membrane, MT1 (*Mel 1a*) and MT2 (*Mel 1b*), consist of seven transmembrane helices, G protein-coupled. Activating G protein signaling, the receptors mediate a wide variety of effects; among others, inhibition of the adenylate cyclase (AC), with a consequent cyclic AMP (cAMP) decrease, regulation of gene transcription, activation of protein kinase C subtypes and changes of intracellular Ca^++^ levels. Independently of receptors, melatonin permeates cell membranes and, owing to its low redox potential, *E*_o_ = −980 mV [21], scavenges the ROS in the cell cytoplasm, mitochondria and nucleus. In the cytoplasm, melatonin maintains GSH homeostasis and interacts with proteins, such as calmodulin (CaCaM), calreticulin and the cytosolic quinone reductase 2 enzyme, (MT3). Melatonin is also a ligand for a nuclear retinoid related orphan nuclear hormone receptor (RZR/RORa) regulating the expression of anti-oxidant enzymes, such as glutathione peroxidase (GPx), glutathione reductase (GRd) and superoxide dismutase (SOD), and downregulating pro-oxidant enzymes, such as the NOSs, particularly the iNOS [35]. Melatonin is accumulated in mitochondria at high concentrations, where it scavenges ROS and RNS. Melatonin also protects cardiolipin from oxidation [36] and prevents respiratory chain complexes, as well as mtDNA from free radical attack, thus ultimately protecting the membrane permeability transition (mPT) pore, thus preventing cell apoptosis.

As schematically represented in Figure 1, over and above the antioxidant redox function, melatonin exerts its hormonal effects via receptor-mediated signaling and activation of specific mRNAs [12]. At least dealing with mitochondria, both the hormonal and the antioxidant function coexist [2,16] and referenced therein [28]. The experimental evidence suggest that the mitochondrial antioxidant activity is more evident at the higher (≥μM) concentrations of melatonin, while the hormonal-like function can be detected at the lower (≤nM) concentrations.

Very recently, using HaCat cells in culture, we have shown that nM melatonin induces the increase of the mRNA expression of neuronal nitric oxide synthase (nNOS) [28]. The mRNA induction was proven to occur within a few hours of cells incubation with melatonin and followed a rise and fall kinetics. The overall process was suggested to be fully compatible with a circadian cycle, mediated by melatonin receptors and with a timing consistent with a nuclear DNA-activated process [28]. The upregulation by melatonin of the nNOS mRNA expression appeared specific, since the eNOS and the iNOS expression was insensitive to melatonin [28]. Interestingly, on a time scale compatible with the nNOS mRNA changes, the cells displayed a lowered ATP_OXPHOS_ production. This finding has been tentatively explained based on the reversible inhibition exerted by the NO on the respiratory chain Complex IV (cytochrome c oxidase, CcOX) [29,30], an event whose pathophysiological meaning is strengthened by the putative existence of a mitochondrial isoform of the nNOS.

In this paper, we report new evidence supporting a melatonin-induced synthesis of the nNOS enzyme occurring within a time scale compatible with the nNOS mRNA, and we show its effect on cell respiration. Over a circadian-compatible time window, we have evaluated the nNOS protein synthesis by HaCat cells incubated with physiological, nanomolar, melatonin. In agreement with previous results [28], we have evaluated those functional parameters, suitable to assess the cell bioenergetic state, correlating them in parallel with changes in composition of the cell culture medium, particularly focusing on the NO end products. The whole picture further suggests that NO chemistry plays a role in the mitochondrial circadian cycle [28,31]. The hypothesis is fully consistent with the finding that the production of nitrite and nitrate is also characterized by a circadian night peak [32] and that the nNOS activity is involved in sleep regulation [33,34].

A bioenergetic involvement of melatonin is supported by the effects induced on the respiratory chain, though their interpretation is not always straightforward [16]. Difficulties in comparing data are partly linked to differences in the experimental set up. Experiments have been carried out, in fact, with fully integrated systems, such as animals or cells in culture, but also using isolated mitochondria. In the latter case, regardless of whether the organelles are functionally intact or not, the nuclear signaling-dependent reactions do not occur. In addition, it should be kept in mind that the intracellular concentration of melatonin putatively reached under the given conditions, unless directly measured, remains uncertain owing to the ability of different cell organelles, e.g., mitochondria [37], and compartments, e.g., nuclei [38], to accumulate melatonin to a different extent.

## 2. Results and Discussion

### 2.1. Experimental Results

The mitochondrial response to melatonin has been investigated using cultured HaCaT cells exposed to increasing amounts of melatonin, from 1 to 100 nM; the activation of nuclear-dependent reactions was also investigated by carrying the experiments within a time scale of hours.

#### 2.1.1. Neuronal NOS (nNOS) Expression and NOx Production

**Figure 2:**
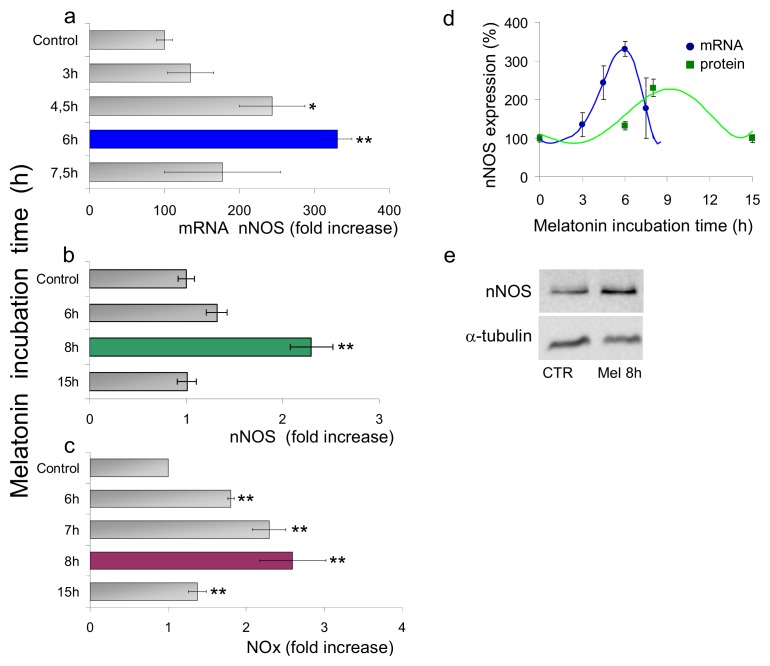
Time-dependent effects of nM melatonin on nNOS expression and NOx production. HaCaT cells were incubated with 1 nM melatonin; at the times indicated, the nNOS mRNA (**a**), nNOS protein (**b**) and nitrite/nitrate (NOx) production (**c**) were assayed. (**a**) QRT-PCR analysis of mRNA expression was performed in the presence of specific primers for nNOS. The relative expression levels were calculated *vs.* untreated controls (ß-actin normalized). The maximal mRNA expression at 6 h (blue). Data +/− SEM, *n* ≥ 3. **p ≤ 0.01 *vs.* control; *p ≤ 0.05 *vs.* control; (**b**) Western blot analysis of nNOS protein. Data shown as fold increase *vs.* the nNOS protein expressed by control cells, as a function of incubation time. The maximal protein synthesis at 8 h (green). Data +/− SEM, *n* ≥ 3. **p ≤ 0.01 *vs.* control; (**c**) NOx accumulation measured in the supernatant as a function of time. Data shown as fold increase *vs.* control (untreated). The maximal NOx production at 8 h (violet). Data +/− SEM, *n* ≥ 5. **p ≤ 0.01 *vs.* control; (**d**) Time-dependent profile of the expression of the nNOS mRNA (blue) and nNOS protein (green); (**e**) Western Blot of cells incubated 8 h with 1 nM melatonin and controls (CTR); α-tubulin as reference.

**Figure 3:**
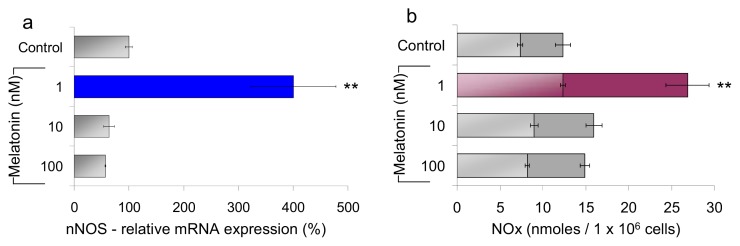
Concentration dependence effect of melatonin (nanomolar) on nNOS expression and NOx production. HaCaT cells were treated with increasing melatonin concentrations (1, 10, 100 nM). (**a**) After 6 h of melatonin incubation, the RNA extracted (10 ng) was retro-transcribed and subjected to QRT-PCR in the presence of specific primers for nNOS. The relative expression levels were calculated *vs.* control after normalization for ß-actin. Data +/− SEM, *n* = 3. **p ≤ 0.01 *vs.* control; (**b**) NO*x* accumulation in the supernatant of HaCaT cells was quantified after 6 h (light bars) and 8 h (heavy bars) incubation with increasing melatonin concentrations. Data are expressed as means +/− SEM, *n* ≥ 5. **p ≤ 0.01 *vs.* control.

Cells were incubated with melatonin (1 nM) for up to ~15 h, and the bioavailability of nitric oxide has been evaluated indirectly by following changes of the cellular NOSs. As shown in Figure 2, the nNOS mRNA level and the nNOS protein expression changed as a function of time, together with the nitrites and nitrates (NOx) concentration, in the culture medium. The nNOS synthesis and the NOx production reach a maximum after ~8 h incubation, *i.e*., ~2 h after cell rising of the nNOS mRNA; noticeably, timing of these processes is consistent with protein synthesis and maturation. The increase of the nNOS mRNA and of the protein concentration both occur in the presence of 1 nM melatonin, falling back to basal levels on a longer time scale, even increasing the concentration of melatonin by orders of magnitude (Figure 3). Interestingly, the maximal effect on the nNOS expression and the NOx accumulation is observed at ~1 nM melatonin, tending to the basal level, with increasing melatonin concentration by one or even two orders of magnitude.

#### 2.1.2. Mitochondrial Respiration and Membrane Potential

**Figure 4:**
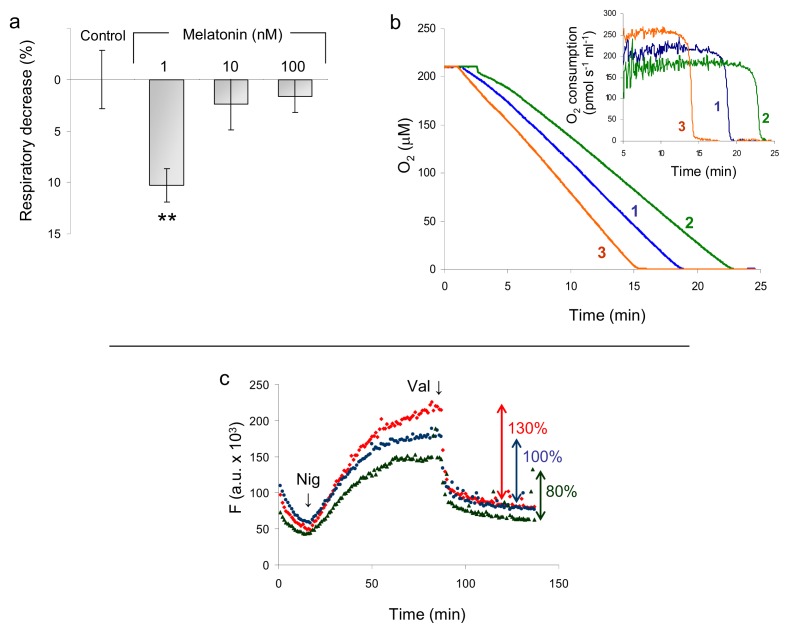
Effect of melatonin on respiration and mitochondrial membrane potential. (**a**) HaCaT cells were incubated 8 h with increasing concentrations of melatonin (1, 10, 100 nM). Respiration was measured and reported as percent of the O_2_ consumption of control HaCaT cells. Cell density: 3.3 × 10^6^ cells/mL; medium: Hank’s containing 1 g/L glucose. Data are expressed as means +/− SEM, *n* ≥ 3. **p ≤ 0.01 *vs.* control; (**b**) Typical O_2_ consumption profiles of melatonin-treated HaCaT cells, in the presence (3, orange) and absence (2, green) of the nNOS inhibitor 7-nitroindazole (7-N); control untreated cells (1, blue). Inset: 1st derivative plot of the traces; (**c**) Mitochondrial membrane potential measured, following JC-1 accumulation, started by the addition to the cells of nigericin 0.6 μM (Nig). Valinomycin (Val) is added (at plateau) to collapse the membrane potential. Excitation wavelength = 490 nm, emission wavelength = 590 nm. Cells maintained in the absence of the NOS substrate arginine (red); control cells (blue). Cells after 8 h incubation with 1 nM melatonin (green).

The spontaneous rate of oxygen consumption was measured amperometrically in intact HaCaT cells respiring on endogenous substrates and incubated 8 h with increasing amounts of melatonin. As shown in Figure 4a, when cells are treated with 1 nM melatonin, a ~10% loss of cell respiration is observed; the effect becomes insignificant at higher melatonin concentrations (up to two orders of magnitude), and it is also reverted by addition of the nNOS inhibitor 7-nitroindazole (7N), (Figure 4b). As shown in Figure 4c, following incubation with melatonin, the mitochondrial membrane potential (ΔΨ) is lowered, by approximately 20%, compared to control. Moreover, accumulation of the probe is maximal when arginine is removed from the cell culture medium, pointing to a correlation between the import of JC-1 and the availability of the NOS substrate.

#### 2.1.3. ATP and Lactate Production

**Figure 5:**
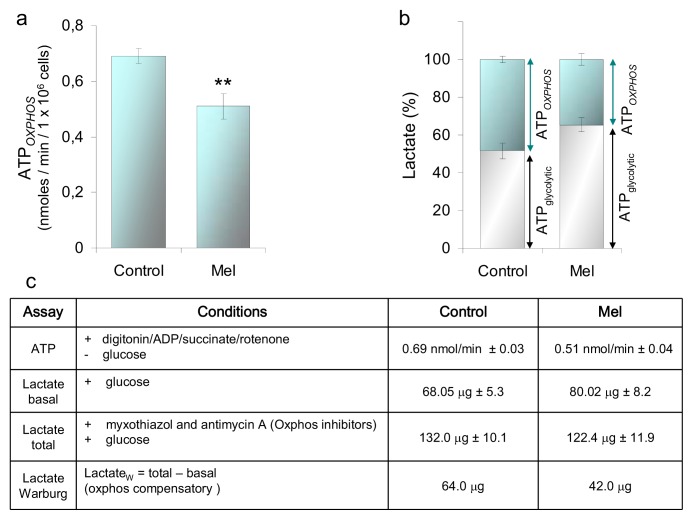
Effect of melatonin on the production of ATP_OXPHOS_ and lactate. ATP and lactate were assayed in HaCaT cells incubated 6h with melatonin, 1 nM. (**a**) Rate of ATP synthesis: Complex II driven ATP synthesis was measured using cells permeabilized with 60 μg/mL digitonin in the presence of 20 mM succinate, 4 μM rotenone and 0.5 mM ADP. ATP measurements carried out according to the luciferin/luciferase assay [41]. Data +/− SEM, *n* ≥ 14. **p ≤ 0.01 *vs.* control; (**b**) Lactate production by HaCaT cells incubated 3 h with glucose, 1 mM, and in the presence and absence of myxothiazol and antimycin A, 10 μM each. Basal lactate (gray bar) is produced by the cells in the absence of inhibitors and has been taken as ≈ ATP_glycolytic_. The lactate produced in the presence of inhibitors has been taken as the maximal lactate (total, 100%). The difference between total and basal lactate is the Warburg lactate (cyan); Warburg lactate ≈ ATP_OXPHOS_. Values are the means +/− SEM; *n* = 4. *p ≤ 0.05 *vs.* control; (**c**) Synoptic table of the ATP and lactate amounts measured under the conditions described in (**a**) and (**b**).

Melatonin increases the nNOS synthesis; hence, the NO bioavailability of HaCaT cells as probed by the accumulation of the NOx. On the same time scale, the cell O_2_ consumption is lowered, with predictable outcomes on the mitochondrial bioenergetics [39,40]. The cell oxidative phosphorylation and the glycolytic efficiency have been, therefore, evaluated, measuring the concentration of ATP and lactate. Compared to controls, the ATP_OXPHOS_ production by the cells incubated with 1 nM melatonin is ~25% lower (Figure 5a), while their glycolytic efficiency, monitored in parallel, is increased (Figure 5b). The lactate produced under basal conditions is indicative of the glycolytic contribution to ATP synthesis (gray bar in Figure 5b). In the presence of myxothiazol and antimycin inhibiting OXPHOS, some additional lactate is produced, due to glycolytic compensation of ATP loss (Warburg effect) (cyan bar in Figure 5b). This Warburg lactate is indicative of the OXPHOS contribution to ATP synthesis. The basal lactate production of the melatonin treated cells is ~65% of the total lactate, to be compared to ~50% of the control cells, whereas the Warburg lactate production is ~48% in the melatonin treated cells and 35% in controls (Figure 5c). Overall, the data strengthen the hypothesis that several hours’ incubation with 1 nM melatonin is able to induce a measurable depression of the oxphos function, compensated by glycolysis.

### 2.2. Discussion

#### 2.2.1. Concentration of Melatonin and Protocol of Administration

Over the years, concentrations of melatonin from ~10^−9^ M up to ~10^−4^ M have been used to investigate the interactions between melatonin and mitochondria [16]. Measurements have been carried out in the presence of the melatonin cell receptors*,* as *in vivo* or *in vitro*, using cultured cells, but also in the absence of the receptors, when using intact mitochondria or sub-mitochondrial particles. To perform *in vivo* experiments, melatonin was injected intra-peritoneally (i.p.) or chronically administered to the animals in drinking water, and their liver or brain mitochondria were isolated and assayed [42–44]. Protocols were compatible with a blood circulating drug concentration, variable [45–47], though somewhat higher, than the ~10^−9^ M physiological one [48].

Alternatively, mitochondria were isolated first and then exposed to variable amounts of melatonin, down to ~10^−9^ M concentrations [49]. In these experiments, a significant specific enhancement of the eT activity of Complex I and Complex IV, was observed and more patently at a concentration of melatonin ≥10^−6^ M. The authors proposed that the increase of activity was related to: (i) optimization of the mitochondrial membrane fluidity, due to melatonin prevention of membrane lipid peroxidation [50]; (ii) direct scavenging of the H_2_O_2_[51,52]; and (iii) stabilization of mitochondrial GSH [53–55].

**Figure 6:**
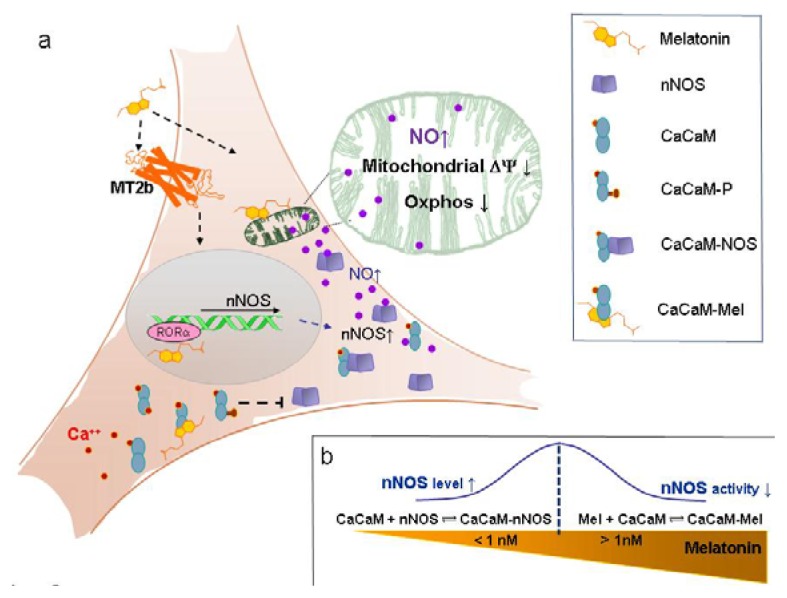
Melatonin and the “keratinocyte hypothesis”. At nanomolar and sub-nanomolar melatonin concentration, a melatonin-receptor-mediated transient nNOS overexpression is triggered. At this stage, calmodulin can predominantly interact with nNOS, leading to production of NO and modulation of mitochondrial function. Upon increasing the external concentration of melatonin (>1 nM) or its time of incubation, its intracellular concentration also rises: under these conditions, melatonin binds/inactivates calmodulin (CaCaM) leading to nNOS inhibition [57,58]. (**a**) Schematic drawing of melatonin traffic and signaling in a keratinocyte (HaCaT cells); (**b**) How the co-existing equilibrium of melatonin with calmodulin and NOS may affect NOS activity. Intracellular melatonin gradient (orange) and nNOS expression/activity (blue).

Somewhat in contrast, Lopez, A., *et al*. [37] have shown that melatonin added to mice liver mitochondria at concentrations from 1 nM to 1 mM is able to decrease respiration in a concentration-dependent manner, by inhibiting the ADP-dependent state 4 to state 3 transition. In the same line, Reyes-Toso [56] observed that melatonin added *in vitro* to mitochondria or chronically administered to the animals in the diet inhibits the substrate-induced (ADP) state 4 to state 3 transition, suggesting that this might protect mitochondria from oxidative damage. Our investigation on HaCaT keratinocytes allowed us to study the effect of nanomolar melatonin, without losing the contribution of the nuclear signaling. Interestingly, only under these conditions, it has been possible to observe the enhancement of the nNOS expression and the depression of mitochondrial activity (Figure 6).

#### 2.2.2. Nanomolar Melatonin, nNOS Synthesis and Involvement of Complex IV

Keratinocytes (HaCat cells), incubated for up to 15 h with nanomolar melatonin, after 6–8 h, display a transient rise of the nNOS and accumulate NOx (nitrite/nitrate) in the culture medium. The nNOS protein synthesis lags behind the rise of the corresponding nNOS mRNA: the protein increases transiently, and within hours, returns back to its basal level; the ~2 h shift between the protein and the mRNA synthesis is consistent with a nuclear DNA-dependent pathway. Following incubation with nanomolar melatonin and on a time scale similar to that of both the nNOS changes and the NOx accumulation, the mitochondrial respiration becomes slightly, but significantly, depressed (~10%). The mitochondrial membrane potential, instead, decreases to a larger extent, by ~20%, suggesting that under these conditions and at least in keratinocytes mitochondria, the mechanism(s) responsible for maintenance of ΔΨ are more affected than respiration [59]. This observation is compatible with the hypothesis that, as a cell responds to melatonin, a small amount of free NO, nanomolar or less [60], is made available in the mitochondrial environment. NO, in fact, is able to rapidly and reversibly inhibit Complex IV in turnover with electrons and oxygen [61], a reaction whose occurrence has been demonstrated at all integration levels of the enzyme, from purified to fully integrated, in intact mitochondria and in cells [39,62,63]. Synchronously with the down regulation of the respiratory chain, we have also observed a decrease of the cell ability to synthesize ATP_OXPHOS_, compensated by an increased production of glycolytic ATP, a behavior originally observed in astrocytes [64], the so-called Warburg effect [65].

Taken together, these findings suggest that nanomolar melatonin administered to intact HaCaT cells transiently activates the nNOS synthesis, with production of NO and reversible inhibition of Complex IV [61,62,66]. This leads, in turn, to a transient metabolic shift towards glycolysis [28]. It is worth recalling, in fact, that depending on the mechanism by which NO reacts with Complex IV, it is possible to detect either a physiological modulation of the electron transfer through the respiratory chain or a more persistent inhibition of mitochondrial respiration [63,67]. Relevant parameters controlling the reaction mechanism were shown to be the cell oxygenation state, the electron flux through the respiratory chain and, indeed, the NO concentration [63,68–70]. The fraction of mitochondria in state 3 and state 4 respiration is also important, as state 3 mitochondria are more prone to Complex IV nitrosylation (persistent inhibition) [71].

Based on independent measurements [68], under normal conditions of cell/tissues oxygenation and supply of mitochondrial reducing substrates, a pulse of NO, limited in extent and time, would lead to formation of a labile Complex IV-NO_2_^−^ derivative. Interestingly, and relevant to the melatonin effects herein described, the activity of Complex IV is promptly recovered upon decreasing the NO bioavailability.

Melatonin, at pharmacological concentrations (10^−6^ M−10^−3^ M), either by signaling (Figure 1) or acting as a radical scavenger, optimizes the eT within the respiratory chain [16] and referenced therein cited, likely minimizing the effects of the NO inhibition of Complex IV. It is tempting to speculate that when the inhibition of Complex IV by NO prevails over the optimization of the electron transfer, then the radical scavenging capacity of melatonin might become insufficient to compensate for inhibition [63]. We suggest that this occurs at ~10^−9^ M melatonin in the extracellular environment, a concentration far too low to be effective in radical scavenging, but apparently ideal to activate the receptor-mediated nNOS chemistry: the result is a transient release of NO with control of both the rate of respiration and the ATP_OXPHOS_ synthesis. As expected, the Warburg effect is also clearly observed and, as a side effect, a higher fraction of O_2_ becomes available for close by cells, the so called O_2_-diversion [72]. Within the limits of a comparison between a cell culture system and an *in vivo* state, we suggest that the events involving 10^−9^ M melatonin and mitochondria are physiological and might occur in a circadian context.

Thus, under the conditions herein used to treat keratinocytes, 1 nM melatonin in the external medium depressed cell respiration and transiently increased the nNOS expression. Both effects were reverted upon increasing the concentration of melatonin or maintaining the cells in the presence of 1 nM melatonin for longer incubation times. The mechanism through which low melatonin concentrations in the extracellular medium might trigger the nNOS expression and, apparently, in a paradoxical biphasic mode, remains unclear. It is tempting to speculate that a time and concentration-dependent feed-back controls the effects of melatonin on the nNOS and that the basis of this control might involve the melatonin-calmodulin interaction and signaling [57].

In this frame, when the extracellular hormone concentration is low (nanomolar or less), the nuclear mediated nNOS activation occurs, also sustained by the cell availability of calmodulin (high affinity nNOS cofactor [73]). As the incubation time increases, the intracellular concentration of melatonin (and/or its metabolites) increases, too (Figure 6b). At this stage, due to the high affinity of melatonin for calmodulin [74] a competition between melatonin and nNOS for calmodulin occurs, inducing progressive nNOS inhibition [58] (Figure 6).

The existence of such equilibrium, if confirmed, would explain the biphasic behavior observed, also reconciling some discrepancies in the literature about the effects of melatonin, both on mitochondria and NOS.

## 3. Experimental Section

### 3.1. Chemicals

Dulbecco’s modified Eagle’s medium (DMEM) and fetal bovine serum (FBS) were from Invitrogen Life Technologies (GIBCO, Paisley, UK) and from PAA (Linz, Austria). Melatonin, JC-1 and all other reagents were from Sigma (St. Louis, MO, USA), unless otherwise specified. Real-time PCR reagents were from Stratagene (Santa Clara, CA, USA).

### 3.2. Cell Culture

HaCaT cells were grown in Dulbecco’s modified Eagle’s medium (DMEM), 10% fetal bovine serum (FBS), containing 4.5 g/L glucose, 0.05 mg/mL gentamycin and 2 mM l-glutamine, in 25 cm^2^ flasks or multi-well plates. Cultures were maintained at 37 °C, under 5% CO_2_ and 95% air. Before melatonin treatment, cells were grown for ~24 h in 1 g/L glucose DMEM, w/o FBS and phenol red. When required, cell lysis was carried out by TRIzol or CelLytic™ M Cell Lysis reagent in the presence of Protease Inhibitor Cocktail (1:100); protein content was determined by the Bradford reaction, and citrate synthase activity was assayed as representative of the mitochondrial mass.

### 3.3. nNOS mRNA Determination

The nNOS mRNA was determined according to the protocol detailed in [28]; briefly, HaCaT cells (~3 × 10^6^ cells) were harvested and total RNA isolated; the reverse transcription was carried out using SideStep™ II QPCR cDNA Synthesis Kit (Agilent Technologies, Santa Clara, CA, USA). The QRT-PCR was performed using a Stratagene Mx3005p System (Agilent Technologies, Santa Clara, CA, USA). All reactions have been carried out in triplicate.

### 3.4. nNOS Detection by Western Blot

HaCaT cells were lysed with CelLytic™ M reagent (Sigma) in the presence of protease inhibitors (Sigma). The proteins were separated on 10% SDS-PAGE gels and transferred on nitrocellulose membranes (Whatman, GE Halthcare UK, Buckinghamshire, UK) 1 h at 100 mA. After 2 h blocking (PBS with 0.1% tween and 3% BSA), the membrane was incubated overnight at 4 °C with primary rabbit polyclonal anti-nNOS antibodies (from BD Transduction Laboratories, Buccinasco, MI, Italy); α tubulin was used as the reference. A secondary ECL TM anti-rabbit antibody HRP (Jackson, Baltimore, PA, USA) was thereafter incubated 1 h at 25 °C and chemiluminescence determined (Amersham, GE Halthcare UK, Buckinghamshire, UK). Densitometric analysis was carried out by the KODAK 1D Image Analysis Software (Eastman Kodak Company, Rochester, NY, USA).

### 3.5. Nitrite/Nitrate (NOx) Determination

Accumulation of the NOx in the culture medium of HaCaT cells (~2.5 × 10^5^ cells/mL) was measured after 6 h and 8 h exposure to melatonin at the given concentrations or at different times of incubation with 1 nM melatonin (see text). The NOx content was determined fluorometrically (Fluorometric Assay Kit, Cayman Chemical Co., Ann Arbor, MI, USA).

### 3.6. Cell Respiration

HaCaT cells, grown overnight in an antibiotic/FBS-free DMEM medium, were incubated 8 h with increasing melatonin concentrations (1, 10, 100 nM); when required, the nNOS inhibitor, 7 N, was added (500 nM) 30 min before the measurement. For the assay, cells were resuspended in Hank’s buffer containing 5.5 mM glucose at a final cell density, 3.3 × 10^6^ cells/mL. Cell respiration was evaluated using high resolution respirometry (2k-Oxygraph OROBOROS Instruments, Innsbruck, Austria).

### 3.7. Mitochondrial Membrane Potential

The mitochondrial H^+^ electrochemical potential gradient of intact cells (ΔμH^+^) was evaluated following the mitochondrial electrophoretic accumulation of JC-1 (Sigma) [75]. In the presence of the ionophore nigericin, converting ΔpH into ΔΨ [76], the fluorescence reaches a maximum, whose level depends upon the membrane potential value. The fluorescence signal is rapidly dissipated by 0.2 μM valinomycin (ΔΨ ≈ 0), thus allowing a Δ*F* to be evaluated (proportional to the membrane potential).

### 3.8. ATP Measurements

The ATP concentration was quantified by chemiluminescence, as described in [28]. Briefly, cells were incubated with 1 nM melatonin for 6 h. The rate of ATP production was evaluated after cell membrane permeabilization with 60 μg/mL digitonin, 20 min at 25 °C; 20 mM succinate and 0.5 mM ADP were thereafter added, in the presence of 4 μM rotenone, to induce ATP synthesis. Measurements were performed using the ATPlite kit (Perkin Elmer, Waltham, MA, USA), on a VICTOR™ Multilabel Counter (Perkin Elmer, Waltham, MA, USA) equipped with 96-well (white) plates.

### 3.9. Lactate Measurements

Cells (~3 × 10^6^ cells), have been incubated 6 h with melatonin, 1 nM, and then, the lactate was measured. In order to energetically synchronize the cells, after the first 2 h, incubation cells were starved 1 h from glucose. Thereafter, glucose, 1 mM, was re-added to the cells for a further 3 h. Lactate determinations have been carried out in the absence of myxothiazol and antimycin A or in their presence (10 μM each) to fully inhibit OXPHOS. The lactate concentration was determined spectrophotometrically on the cell supernatant collected by centrifugation (1000 × *g*, at 4 °C, 10 min).

### 3.10. Statistics

The number of independent measurements is indicated in the figure legend. Significance was determined using the Student *t*-test, run by Excel (Microsoft Windows platform). The error bars correspond to the standard error of the mean (SEM); all *p*-values correspond to two-sided sample *t-*test, assuming unequal variances. A *p*-value ≤ 0.05 was considered significant.

## 4. Conclusions

Based on our results, the physiological meaning of the effects induced by melatonin on the nNOS, and, thereby, on cell bioenergetics, is purely hypothetical, demanding further assessment and independent evaluation. All together, the experimental evidence points to two mechanistically-independent activities exerted by melatonin at the mitochondrial level. The first one is based on the antioxidant radical scavenging properties of melatonin: this is best observed at a high concentration of melatonin (≥10^−6^ M); the second one is more consistent with a hormonal-like function and appears to be nuclear-dependent, possibly receptor-mediated, thus requiring a suitable time of cell incubation with nanomolar amounts of melatonin. Interestingly enough, these extracellular concentration values are compatible with those reached in our body during night peak, ~100–200 pg/mL [48,77], *i.e*., ~1 nM melatonin. We believe that under these conditions, the intra-cellular concentration of melatonin increases gradually and allows its accumulation in the different cell compartments. In this respect, it is worth mentioning that the evaluation of the melatonin activity at the mitochondrial level can be particularly difficult *in vivo*, if we consider that: melatonin can be imported and accumulated in the mitochondrion [37], while metabolites of melatonin retain a substantial bioactivity [45]. Furthermore, calmodulin, in the cell, is in equilibrium with both melatonin and nNOS, indeed, among other targets: this peculiar state should be considered when addressing the melatonin effects on mitochondria. With these premises, the comparison between data obtained using different experimental approaches may not be straightforward. In conclusion, also based on the literature, our data suggest that melatonin controls mitochondrial efficiency at different levels, by: (i) favoring the eT through the respiratory chain, in a concentration-dependent manner and independently on nuclear-DNA-mediated reactions; while (ii) causing a mild transient inhibition of Complex IV, with a mitochondrial glycolytic shift, an effect mediated by nuclear signaling in a circadian compatible time-window.
